# Toward intraoperative tissue classification: exploiting signal feedback from an ultrasonic aspirator for brain tissue differentiation

**DOI:** 10.1007/s11548-022-02713-0

**Published:** 2022-08-04

**Authors:** Niclas Bockelmann, Daniel Schetelig, Denise Kesslau, Steffen Buschschlüter, Floris Ernst, Matteo Mario Bonsanto

**Affiliations:** 1 Institute for Robotics and Cognitive Systems, University of Lübeck, Ratzeburger Allee 160, 23562 Lübeck, Germany; 2 Söring GmbH, Justus-von-Liebig-Ring 2, 25451 Quickborn, Germany; 3 Department of Neurosurgery, University Hospital Schleswig–Holstein, Ratzeburger Allee 160, 23538 Lübeck, Germany

**Keywords:** Tactile sensor, Tissue differentiation, Machine learning, Convolutional neural network, Ultrasonic aspirator

## Abstract

**Purpose:**

During brain tumor surgery, care must be taken to accurately differentiate between tumorous and healthy tissue, as inadvertent resection of functional brain areas can cause severe consequences. Since visual assessment can be difficult during tissue resection, neurosurgeons have to rely on the mechanical perception of tissue, which in itself is inherently challenging. A commonly used instrument for tumor resection is the ultrasonic aspirator, whose system behavior is already dependent on tissue properties. Using data recorded during tissue fragmentation, machine learning-based tissue differentiation is investigated for the first time utilizing ultrasonic aspirators.

**Methods:**

Artificial tissue model with two different mechanical properties is synthesized to represent healthy and tumorous tissue. 40,000 temporal measurement points of electrical data are recorded in a laboratory environment using a CNC machine. Three different machine learning approaches are applied: a random forest (RF), a fully connected neural network (NN) and a 1D convolutional neural network (CNN). Additionally, different preprocessing steps are investigated.

**Results:**

Fivefold cross-validation is conducted over the data and evaluated with the metrics *F*1, accuracy, positive predictive value, true positive rate and area under the receiver operating characteristic. Results show a generally good performance with a mean *F*1 of up to 0.900 ± 0.096 using a NN approach. Temporal information indicates low impact on classification performance, while a low-pass filter preprocessing step leads to superior results.

**Conclusion:**

This work demonstrates the first steps to successfully differentiate healthy brain and tumor tissue using an ultrasonic aspirator during tissue fragmentation. Evaluation shows that both neural network-based classifiers outperform the RF. In addition, the effects of temporal dependencies are found to be reduced when adequate data preprocessing is performed. To ensure subsequent implementation in the clinic, handheld ultrasonic aspirator use needs to be investigated in the future as well as the addition of data to reflect tissue diversity during neurosurgical operations.

## Introduction

Microsurgical resection is the standard treatment for a majority of tumors in the central nervous system. Survival rate and life quality depend, among other factors, on the extent of resection [1–3]. In modern neurosurgery, tumor localization is performed using MR- and/or CT-imaging and neuro-navigation systems. To date, however, tumor margins are difficult to recognize intraoperatively, since neuro-navigation systems can lose precision during surgery due to cerebrospinal fluid loss and partial tumor removal (i.e., brain shift). Inaccurately identified tumor margins can lead to tumor remnants or healthy brain tissue being unnecessarily resected. If the tumor resection is performed near functional brain areas (i.e., somatosensory cortex, language-relevant brain areas (Broca/Wernike)), this may result in permanent damage to the patient such as paralysis and speech disorders. For further intraoperative support, systemically administered fluorescent dyes can be used to enhance tumor visibility. For this purpose, dedicated light sources are integrated into the operation microscope. Unfortunately, not all brain tumor entities can be enriched with fluorescent dyes [4–7]. Alternative applications in neurosurgery include the use of intraoperative 3D-ultrasound and intraoperative MR-/CT-imaging, both logistically complex to operate with [8–12]. Further, new methods to visualize brain tumor tissue are, e.g., optical coherence tomography (OCT) or Raman histology, which are usually associated with a restructuring of the operating workflow [13–17]. Other studies indicate that healthy brain and tumor tissue differ from each other based on their mechanical properties, such as elasticity [18–24]. This difference can often be palpated by a highly trained neurosurgeon and is subject to the current research in which system behavior of tactile sensors is used to indicate tissue properties. To estimate tissue properties quantitatively, Tanaka et al. [25] utilized a biocompatible balloon-based sensor system, which is expanded by fluids. The expansion of the balloon while in contact with tissue allows to draw conclusions about tissue properties to some extent. Tissue discrimination was investigated on urethane gels as well as on healthy white and gray matter of porcine brain tissue. In the work of Johannsmann et al. [26], a piezoelectric tactile sensor with torsional resonators was investigated for tissue differentiation capabilities. Resonance frequency and bandwidth shifts of the sensor between contact and non-contact states are examined and correlated with healthy and tumorous brain tissue in in vivo rat experiments. Similarly, piezoelectric bimorph sensors are used in Stroop et al. [27] to distinguish ex vivo porcine brain tissue regions such as cortex surface, white matter, basal ganglia and thalamus. The tactile sensor is excited using multisine for obtaining frequency response functions, which are processed and clustered subsequently using k-means algorithm. Even though these approaches are showing promising results, they (among multiple others) are relying on newly designed instruments that always require a change in the surgical workflow and are typically intended for robotic or endoscopic applications only [25–31].

Since the early 1980s, a commonly used neurosurgical instrument for tumor removal is the ultrasonic aspirator [32, 33]. This instrument combines three functions simultaneously:

1. Ultrasonic-based fragmentation of tissues—mechanical oscillation of a hollow tube causes tissue ablation
2. Aspiration—generation of negative pressure allows removing ablated tissue through a hollow tube
3. Irrigation—addition of saline solution regulates the liquid content and enhances tissue aspiration.

Each of these functions can be set by the user of the ultrasonic aspirator independently to ensure efficient tissue fragmentation.

**Fig. 1:**
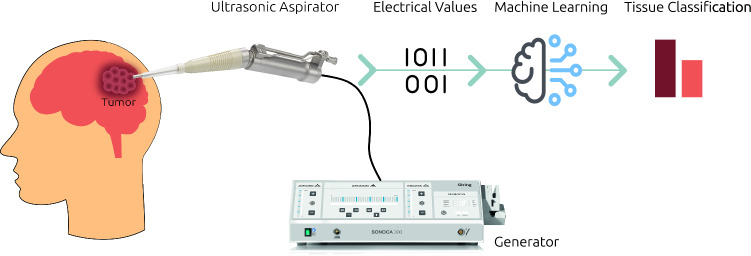
Conceptual representation of tissue differentiation using an ultrasonic aspirator. Electrical data that are recorded during tissue interaction with an ultrasonic aspirator are processed with machine learning algorithms to infer information about tissue properties

Since the electrical system of the ultrasonic aspirator shows load-dependent behavior, it is expected that information regarding tissue properties can be extracted using the electrical data of the generator from the physical interaction with tissue [34]. This behavior can be exploited to establish a relationship between the electrical values and the tissue properties using machine learning methods. A schematic representation of the entire pursued process is shown in Fig. 1: during the application of the ultrasonic aspirator to remove malignant brain tissue, the electrical data coming from the generator are recorded. These electrical data are subsequently processed by machine learning methods, which allow a classification of the currently examined tissue. This information could then be redirected to the surgeons as a support system. Thus, on the one hand, an increase in patient safety could be ensured and, on the other hand, the inexperience of new surgeons could be compensated. Furthermore, the workflow can be improved through the combination of therapy and diagnostics in one device. In the previous work, we showed that it was possible to distinguish synthetic tissue models from another with a non-resection mode of the generator in a first proof of concept [34]. However, since such a mode does not reflect the clinical case, the behavior of the electrical values during tissue fragmentation must be analyzed. For the first time, this work investigates whether tissue differentiation is possible during fragmentation of tissue material in a laboratory setting.

**Fig. 2:**
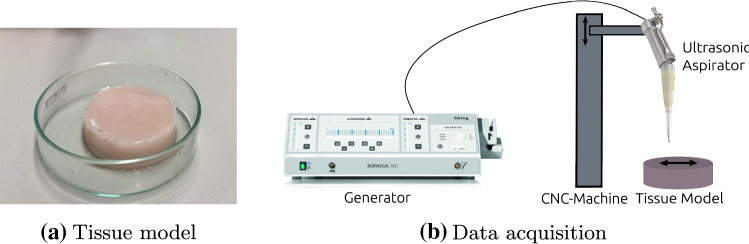
Tissue model and data acquisition process. Example of artificial tissue model is shown in **a**. Schematic representation of data acquisition process is shown in **b**. Tissue ablation and data acquisition are performed using a CNC-machine that traverses the ultrasonic aspirator in lanes across the artificial tissue model

## Materials and methods

### Data acquisition

Acquisition of a large database of tumorous and healthy brain tissue, utilizable for data analysis and training, is very time-consuming and difficult. Especially the wide variety of ‘experimental settings’ in a medical context (environmental variables, surgeons, etc.) make constant and high-quality data collection difficult and requires data collection over multiple years. We, therefore, employed artificial tissue models to acquire data in a controlled manner with consistent experimental settings. The data are collected on our tissue models, which resemble the same mechanical properties as brain tissue and can be synthesized to allow for a wide range of mechanical properties.

For this study, tissue models are created with two different mechanical properties, resembling healthy porcine brain tissue and human meningioma [35]. Such an artificial tissue model is shown in Fig. 2a. These tissue models are of homogeneous consistency and provide an initial basis for this proof-of-concept study. Qualitatively speaking, these can be categorized in terms of tactile sensation as *very soft* (meningioma) and *soft* (healthy brain tissue). These categories *very soft* and *soft* are used as classification target in the subsequent experiments.

To further ensure a controlled environmental setting, we employed a CNC machine (three degrees of freedom: translational movement in *x*-, *y*- and *z*-axes) for tissue ablation and subsequent data acquisition: Typically, an ultrasonic aspirator is used as a handheld device. However, manual usage is naturally associated with multiple sources of uncertainty (e.g., movement speed), which makes experiments difficult to replicate. Therefore, we performed data acquisition using a CNC machine, which is programmed to traverse in lanes over the tissue model, while ablation is performed. For this process, the ultrasonic aspirator is mounted with a clamp system in the CNC machine, which then guides the device. Care is taken when traversing the lanes to maintain sufficient distance from the edge of the tissue samples so as not to provoke edge effects that could result in stiffness changes. This data acquisition procedure (schematically depicted in Fig. 2b) allows to determine exactly when contact is established with the tissue. While data acquisition of handheld ultrasonic aspirators will be part of further studies, this experimental setup serves as a first step in understanding the recorded data as well as classification possibilities and thus provides a defined, reproducible framework, well suited for this feasibility study.

The traversing lane pattern of the CNC machine consists of three phases: (1) the ultrasonic aspirator is positioned above the tissue model and is brought into contact with the tissue by descending; (2) when contact is established, the ultrasonic aspirator traverses back and forth once along a predefined path length; (3) the ultrasonic aspirator detaches from the tissue and returns to its starting position. These three phases are referred to as the initial idle phase, contact phase and final idle phases. A total of five paths are traversed for each tissue model. In order to avoid loss of contact during ablation, the CNC machine continuously moves into the depth of the tissue until a maximum depth of 1.4 mm is reached at the end of the process. One data recording entails the fragmentation process on one lane of the tissue model. In all three phases of one data recording, electrical data are recorded with a sampling frequency of 1000 Hz. A total of nine different electrical features are recorded, which include standard features like, e.g., voltage, current and frequency.

**Fig. 3:**
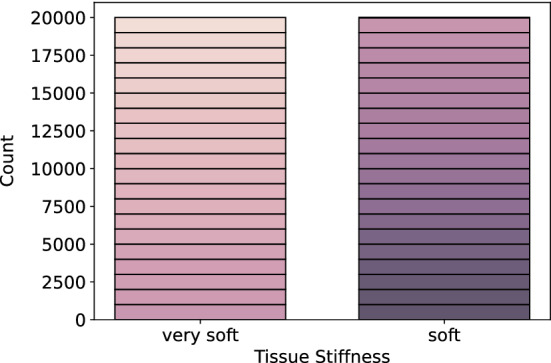
Distribution of measurement points over two classes *very soft* and *soft*. Each differently colored box indicates data from one recording

For this feasibility study, we chose a generator setting (ultrasound: 11%, aspiration: 160 mbar, irrigation: 7 ml/min), which is typically used and well suited for *very soft* and *soft* materials. In total, four tissue models are used per stiffness class, which leads to 40 recordings that are conducted with a contact phase duration of 4.5 s. Since a recording is performed on the same homogeneous tissue model, no significant changes of the signals are to be expected during a recording. Additionally, we want to have direct feedback in later clinical use and therefore want to train the system so that only a small amount of time is required to allow classification. Thus, only the first second of the contact phase is used, leading to 1000 measurement points per recording. This results in a total of 40,000 measurement points that are processed and classified individually (hereinafter referred to as sample-based classification) with respect to their tissue stiffness classes *very soft* and *soft*. In Fig. 3, the distribution of the measurement points is shown over the two classes. It can be seen that the data are well distributed with 20,000 measurement points per class, each consisting of 20 recordings—indicated by the differently colored boxes. All data are sampled with a sampling frequency of 1000 Hz.

### Classification models

Classification of the different tissue models based on the electrical features of the ultrasonic aspirator is performed with three different classification algorithms. Firstly, a random forest (tree size: 100, feature importance: Gini index) is used a baseline model [36]. This algorithm is chosen because it provides a robust baseline with its simple and comprehensible design.

**Fig. 4:**
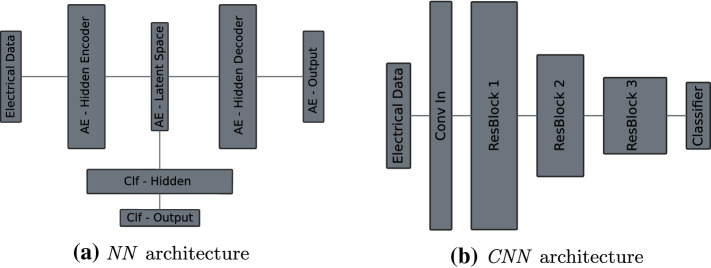
Network architectures. Detailed information about hyperparameters can be found in appendix in Tables 3 and 4. *AE* autoencoder, *Conv* convolution, *Clf* classifier, *ResBlock* residual block

The second algorithm is implementing a neural network (NN) structure with a combination of an autoencoder and a classifier network. The autoencoder is used for regularization purposes to enforce the NN to only learn useful properties of the data in the latent space. Finally, to classify the data, a classification network is used, whose input is based on the latent space. Both the autoencoder and classification network are trained simultaneously end to end with a combined loss inspired by Santilli et al. [37]. The combined loss is defined as:

$$\begin{aligned} \text {loss} = \text {loss}_\text {clf} + \alpha \times \text {loss}_\text {ae} \end{aligned}$$

with $\text {loss}_\text {clf}$ as the cross-entropy loss of the classification network and $\text {loss}_\text {ae}$ as the mean-squared error loss of the autoencoder. The effect of the $\text {loss}_\text {ae}$ on the combined loss is controlled by the weight parameter $\alpha$. Throughout the experiments, $\alpha$ is set to 0.3, which was empirically determined by us to be a good general fit. The autoencoder consists of one fully connected hidden layer of size 32 in the encoder and decoder parts with one latent space layer of size nine in between. The classification network features one hidden layer of size four as well as one output layer. A visual representation of the network can be found in Fig. 4a, while Table 3 provides a more detailed overview of the used parameters.

Both classifiers, the RF and NN, work on a sample-based level; thus, no temporal influences of the signals are leveraged. In order to incorporate temporal information into the training process, a deep convolutional neural network (CNN) with a sliding-window approach is used as a third classification algorithm. The CNN uses a 1D residual network architecture that allows an ease of training due to residual blocks with identity skip-connections [38, 39]. A total of seven convolutional layers are used, formed by three residual blocks. With the beginning of every residual block, the numbers of feature channels are doubled, while a stride size of two is applied—with an exception for the second residual block in which the feature channel number is not doubled. The kernel size is fixed to seven on all layers, and the number of the first feature channels is set to 16. Every convolutional layer is followed by a 1D batch normalization layer. After the last convolutional layer, an average global pooling is performed to enable a transition to the fully connected output layer. The input length to the CNN is based on the window that is slide over the signal with a size of 64. Prediction is always performed on the last value in the window and only done if 63 previous values are available. Cross-entropy loss is chosen as the loss function for this architecture. An overview of the architectural design is provided in Fig. 4b with its accompanied parameter depiction in appendix in Table 4.

Training for both neural network approaches is done with Adam optimization [40] using an initial learning rate of 0.01, which is multiplied by a factor of 0.1 upon reaching a plateau on the training data. Early stopping is implemented based on validation data performance with an otherwise maximum epoch count of 2000. The batch size is set to 16,384, and a weight decay of 0.001 is applied. Input data are normalized with zero mean and unit variance. In order to manage overfitting, dropout is used in case of NN in the hidden layers of the encoder and classification networks, as well as after every convolutional layer in the CNN. In both cases, the dropout value is set to 0.3. Furthermore, for the NN a Gaussian noise of 0.1 is added to the input features of the training data to prevent overfitting. During inference on the NN, the decoder part of the autoencoder is omitted and only the classification output is used.

### Experiments

The experiments are carried out in two steps: Firstly, the performance of the aforementioned machine learning approaches is evaluated; secondly, the influence of data preprocessing on classification is analyzed:

All experiments are conducted and analyzed over a fivefold cross-validation. It is ensured that the splits do not divide the recording data, such that data from the same recording are not found in training and test sets. For the neural network-based classifiers, an additional split of 87.5% and 12.5% is performed for training and validation data, respectively, to prevent overfitting. Different metrics are recorded on the test data for evaluation purposes: the *F*1-score (*F*1), accuracy (ACC), positive predictive value (PPV), true-positive rate (TPR) and the area under the receiver operating characteristic (AUROC).

As a first experiment, the performance of the three presented classification methods, RF, NN and CNN is evaluated without any prior signal processing. All methods are provided with the same electrical input signals, from which a differentiation of the tissue models between *very soft* and *soft* is then to be carried out.

**Fig. 5:**
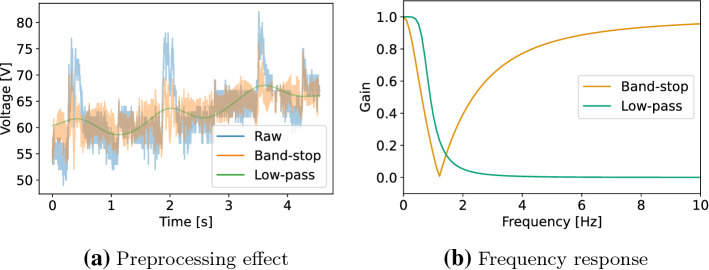
Preprocessing filters. Effect of preprocessing on voltage signal during contact phase is shown in **a**. Frequency response of preprocessing filters is shown in **b**. Raw signal with noise in blue, band-stop filtered signal and frequency response in orange, low-pass filtered signal and frequency response in green

The second experiment concerns possible data preprocessing: During data analysis, a significant portion of recordings show a superimposed noise signal with a frequency of around 1.2 Hz, possibly caused by periodic behavior of the irrigation function of the instrument, which could impede classification performance. Two different preprocessing approaches are applied to filter this noise: a low-pass filter using a Butterworth filter, leading to a smooth signal, and band-stop filter that uses a notch filter allowing the data to keep high-frequency components. The contact phase of one recording is shown in Fig. 5a, which illustrates the influence of the two preprocessing approaches on the raw signal with periodic noise. The design choices of the two filters were determined empirically to reduce the occurrence of noise at 1.2 Hz. The low-pass Butterworth filter was set to a third-order filter using a cut-off frequency of 0.75 Hz and a sampling frequency of 1000 Hz. The band-stop notch filter’s setting showed an overall sufficient result using a quality factor of 0.4 with the frequency to remove set to 1.2 Hz at a sampling frequency of 1000 Hz. A visual representation of the filters can be found as frequency response plots in Fig. 5b. The influence of the two preprocessing filters is evaluated with regard to the classification performance of RF, NN and CNN.

## Results and discussion

### Classification performance

**Table 1:** Results of different classifiers using raw input without preprocessing

Classifier	*F*1	ACC	PPV	TPR	AUROC
RF	0.688 (0.206)	0.692 (0.203)	0.700 (0.212)	0.692 (0.203)	0.790 (0.220)
NN	0.682 (0.181)	0.687 (0.175)	0.689 (0.182)	0.687 (0.175)	0.735 (0.211)
CNN	0.720 (0.122)	0.735 (0.104)	0.773 (0.097)	0.735 (0.104)	0.778 (0.224)

Metrics provided as mean (standard deviation)

Table 1 presents the results on the classification performance of the three proposed methods. The sample-based approaches RF and NN both show equivalent performance with a mean *F*1 of about 0.68. A slightly higher mean *F*1-score of 0.72 is obtained with the CNN approach. However, reviewing the standard deviations for all five metrics indicates that differences in metrics might be negligible. This is unexpected, as the additional information of the temporal data in the CNN could provide an advantage.

**Fig. 6:**
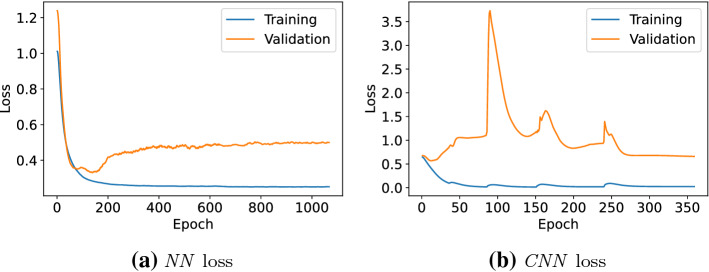
Training and validation loss of NN and CNN on raw data for an exemplary fold

The loss curves of an exemplary fold for the two neural network approaches are shown in Fig. 6. It can clearly be seen that overfitting on the training data presents an issue in both applied methods and can be observed throughout all experiments. This is why multiple countermeasures—mentioned in the classification models in Sect. 2.2—are implemented to keep the effects at a minimum. This high variance can be improved in the future with an increase in the size of the database. The spikes in the validation loss of the CNN are most likely caused by mini-batch gradient descend, a relatively small validation set size and the usage of batch normalization layers. In case of the NN validation loss, this is not observable since the loss is further regularized by the autoencoder part.

**Table 2:** Results on different preprocessing filters

Classifier	Filter	*F*1	ACC	PPV	TPR	AUROC
RF	Low-pass	0.788 (0.238)	0.795 (0.229)	0.805 (0.232)	0.795 (0.229)	0.881 (0.170)
RF	Band-stop	0.658 (0.201)	0.664 (0.199)	0.681 (0.214)	0.664 (0.199)	0.748 (0.245)
NN	Low-pass	0.900 (0.096)	0.902 (0.092)	0.918 (0.070)	0.902 (0.092)	0.935 (0.090)
NN	Band-stop	0.703 (0.199)	0.710 (0.190)	0.714 (0.197)	0.710 (0.190)	0.754 (0.232)
CNN	Low-pass	0.828 (0.192)	0.838 (0.173)	0.849 (0.159)	0.838 (0.173)	0.838 (0.291)
CNN	Band-stop	0.799 (0.146)	0.801 (0.144)	0.816 (0.147)	0.801 (0.144)	0.844 (0.227)

Metrics provided as mean (standard deviation)

### Preprocessing

Two preprocessing filters (Fig. 5a) are applied to handle occurring periodic noise by smoothing the signal with a low-pass filter or by explicitly filtering the corresponding frequency using a band-stop filter. The results are provided in Table 2. In general, the use of both preprocessing approaches leads to an improvement of the classification results. In the best case, the performance of the NN is increased from 0.682 ± 0.181 mean *F*1 on raw data to a mean *F*1 of 0.900 ± 0.096 using a low-pass filter. The noise apparent in the raw data thus seems to have an influence on the quality of the classification, which could be attributed to the wide range of signal values, as exemplified in the noise in Fig. 5a.

With regard to the two filters, we can clearly observe that low-pass preprocessing leads to superior results in direct comparison with band-stop preprocessing. This could be due to the reduced complexity of the data space by smoothing out signals through which the learning of the classification models is simplified and more robust. Concerning only the low-pass filter, the metric is boosted by at least 10 percentage points across all methods with respect to the results based on raw data input. In the case of band-stop filtering, a maximum increase of 5 percentage points can be observed, with a slight deterioration of the metrics for RF.

With respect to the CNN results that include temporal information into the predictions, it can be seen that a slightly increased performance can be achieved with the low-pass-filtered data, although it does not contain high frequencies compared to the band-stop-filtered data. Even though with data being sequential in its recording, the results indicate that the temporal information is not a relevant factor for the classification task at hand and can be improved with adjusted data filtering using a sample-based method such as the NN by a large margin.

Using preprocessing, RF performance is inferior to both neural network-based approaches. Since filtering has simplified the data space, it is arguably easier for the neural network-based approaches to train and perform an improved feature extraction. This feature extraction may be the reason for the difference between the approaches, as the RF classifier is limited to the fixed hand-selected features.

Overall, we can conclude that data preprocessing is essential for improving classification performance. Otherwise, the observed noise impairs results of the different applied methods. Nevertheless, we note that the currently used filtering methods are applied on an entire signal after acquisition and cannot be applied in a real-time setting. Future work will need to address those specific limitations.

## Conclusion and outlook

In this paper, first steps toward intraoperative differentiation of brain tissue using an ultrasonic aspirator were taken: We demonstrated in a laboratory setting that electrical signals, which were acquired during tissue fragmentation, can be used to successfully distinguish tissue models with brain typical stiffnesses, reflecting healthy and tumorous brain tissue. Three different classification methods were evaluated on fivefold cross-validation, yielding a mean *F*1-score of up to 0.900 ± 0.096 with a sample-based neural network classifier. Different preprocessing methods were investigated and were shown to improve the performance by at least 10 percentage points using low-pass filters. Temporal and high-frequency component features were not found to have substantial impact on the classification performance if adequate data preprocessing is conducted. Additionally, the results highlighted the advantage of using the feature extraction capabilities of a neural network in comparison with predefined features in random forest classifiers for this specific classification task.

In order to move further toward clinical real-world data, the problem scenarios will be addressed in smaller steps: In a first step, we already showed the possibility to differentiate synthetic tissues without tissue fragmentation [34]. The second step, considered in this study, deals with the investigation of the feasibility of tissue differentiation under ablation conditions. Future next steps will include the introduction of inhomogeneities in terms of tissue properties and the examination of clinical tissue samples. Additionally, further investigations must be conducted regarding real-time capable signal filtering as well as the effects in handheld ultrasonic aspirator use. Furthermore, a larger database of different tissue properties needs to be created to reflect the variation of tumor entities that are found in clinical practice.

## Appendix A: Hyperparameter

The hyperparameter used to design the NN and CNN architecture shown in Fig. 4 is provided in Tables 3 and 4, respectively.

**Table 3:** Hyperparameters of neural network architecture

	Layer type	Size	Nonlinearity
AE—hidden encoder	FC	32	ReLU
AE—latent space	FC	9	None
AE—hidden decoder	FC	32	ReLU
AE—output	FC	*n*	None
Clf—hidden	FC	4	ReLU
Clf—output	FC	1	None

*n* number of input features, *AE* autoencoder, *Clf* classifier, *FC* fully connected

**Table 4:** Hyperparameters of convolutional neural network architecture

	Layer type	# Kernel	Kernel size	Stride	Nonlinearity
Conv In	Conv	16	7	1	ReLU
ResBlock 1	Conv	32	7	2	ReLU
	Conv	32	1	2	ReLU after addition
	Conv	32	7	1	ReLU after addition
ResBlock 2	Conv	32	7	2	ReLU
	Conv	32	1	2	ReLU after addition
	Conv	32	7	1	ReLU after addition
ResBlock 3	Conv	64	7	2	ReLU
	Conv	64	1	2	ReLU after addition
	Conv	64	7	1	ReLU after addition
Pooling	Adaptive Avg. Pool	–	–	–	–
Classifier	FC	2	–	–	None

Every Conv layer is followed by a batch normalization. The outputs of the last two Conv layers within a ResBlock are added and subsequently processed by ReLU

*Conv* convolution, *FC* fully connected, *ResBlock* residual block
